# A Novel *bHLH* Transcription Factor Involved in Regulating Anthocyanin Biosynthesis in Chrysanthemums (*Chrysanthemum morifolium* Ramat.)

**DOI:** 10.1371/journal.pone.0143892

**Published:** 2015-11-30

**Authors:** Li-li Xiang, Xiao-fen Liu, Xue Li, Xue-ren Yin, Donald Grierson, Fang Li, Kun-song Chen

**Affiliations:** 1 College of Agriculture & Biotechnology, Zhejiang University, Zijingang Campus, Hangzhou, 310058, PR China; 2 Zhejiang Provincial Key Laboratory of Horticultural Plant Integrative Biology, Zhejiang University, Zijingang Campus, Hangzhou, 310058, PR China; 3 The State Agriculture Ministry Laboratory of Horticultural Plant Growth, Development and Quality Improvement, Zhejiang University, Zijingang Campus, Hangzhou, 310058, PR China; 4 School of Biosciences, University of Nottingham, Sutton Bonington Campus, Loughborough, LE12 5RD, United Kingdom; Key Laboratory of Horticultural Plant Biology (MOE), CHINA

## Abstract

Chrysanthemums (*Chrysanthemum morifolium* Ramat.) exhibit a variety of flower colors due to their differing abilities to accumulate anthocyanins. One *MYB* member, *CmMYB6*, has been verified as a transcription regulator of chrysanthemum genes involved in anthocyanin biosynthesis; however, the co-regulators for *CmMYB6* remain unclear in chrysanthemum. Here, the expression pattern of *CmbHLH2*, which is clustered in the IIIf bHLH subgroup, was shown to be positively correlated with the anthocyanin content of cultivars with red, pink and yellow flower colors, respectively. *CmbHLH2* significantly upregulated the *CmDFR* promoter and triggered anthocyanin accumulation when co-expressed with *CmMYB6*. Yeast one-hybrid analyses indicated that *CmbHLH2* was able to bind directly to the *CmDFR* promoter. Moreover, yeast two-hybrid assays indicated protein-protein interaction between *CmbHLH2* and *CmMYB6*. These results suggest that *CmbHLH2* is the essential partner for *CmMYB6* in regulating anthocyanin biosynthesis in chrysanthemum.

## Introduction

Chrysanthemum (*Chrysanthemum morifolium* Ramat.) is one of the most popular ornament plants in the world. Flower color is an important trait for its commercial value. In general, there are three classes of pigments that contribute to flower color: flavonoids, carotenoids and betalains [1]. Anthocyanins, which are derived from the phenylpropanoid pathway by a series of enzymes [2], are the most conspicuous class of flavonoids, which not only contribute to flower color but are also important in attracting pollinators, aiding seed dispersal and protecting plants from UV irradiation damage [3,4]. Studies on the molecular mechanism regulating anthocyanin biosynthesis have shown that the structural genes encoding the enzymes catalyzing anthocyanin biosynthesis are expressed synergistically, regulated by transcription factors, during anthocyanin accumulation [5–7]. Many results indicate *MYBs* play a crucial role in controlling the spatial and temporal expression of anthocyanin biosynthetic genes [8–10].

Recent developments have led to the isolation of chrysanthemum anthocyanin biosynthetic genes [11–16] and their use in the modification of flower colors [11, 17–18]. However, the transcriptional regulatory mechanisms have rarely studied. Hong et al. [16] identified three *CmMYBs* and one *CmbHLH* (basic helix-loop-helix) as the candidate transcription factors for anthocyanin biosynthesis mainly based on clustering analysis. In our previous studies, *CmMYB6* was verified as being involved in the transcriptional regulation on *CmDFR* (Dihydroflavonol 4-reductase) and transient over-expression of *CmMYB6* and *MrbHLH1* (a known anthocyanin co-regulator in *Myrica rubra*) could trigger anthocyanin accumulation in tobacco leaves [10, 19]. This suggested that *CmMYB6* controlled anthocyanin biosynthesis in chrysanthemum flowers in conjunction with endogenous *bHLH* member(s), which led the present research to find the putative anthocyanin related *bHLH* in chrysanthemum.

The basic helix-loop-helix (bHLH) proteins are a superfamily of transcription factors which contain a basic region consisting of 15–17 amino acids that are essential for DNA binding, and a helix-loop-helix domain that is important for formation of homodimers or heterodimers [20]. The *bHLH* transcription factors regulate numerous metabolic processes in plants such as photomorphogenesis [21], fate of epidermal cells [22], metal homeostasis [23, 24] and the flavonoid pathway [5] and their roles in flavonoid, especially anthocyanin, metabolism has been well illustrated in previous research [5–7]. The first *bHLH* transcription factors regulating the flavonoid pathway, *R* and *B*, were identified in maize in 1989 [25], then *An1* was verified in petunia [26], and *AtTT8* in *Arabidopsis* [27]. Subsequently, the *bHLH* member was identified and shown to participate in anthocyanin biosynthesis regulation in dahlia [28] and Asiatic hybrid lily [29]. Recently, Hsu et al. [9] identified three *PeMYBs* responsible for the floral pigmentation patterning in *Phalaenopsis* that interact with endogenous *bHLH* members *PebHLH1-3*. These results suggested that together with *MYB*, the *bHLHs* are essential transcription factors involved in anthocyanin biosynthesis in most plants.

In the present research, in order to investigate regulatory roles of *CmbHLH* in anthocyanin biosynthesis, *CmbHLH2* and a previous reported stress related *CmbHLH1* [30] were isolated, their expression patterns studied. The transcriptional regulatory role of *CmbHLH* was investigated and Yeast one- and two-hybrid assays were utilized to study interactions between *CmbHLH2*, *CmMYB6* and target gene.

## Materials and Methods

### Plant materials

The ray florets of three chrysanthemum (*Chrysanthemum morifolium* Ramat.) cultivars, named ‘Zaoxiaoju no.1’ (‘Z1’), ‘Zaoxiaoju no.2’ (‘Z2’) and ‘Zaoxiaoju no.3’ (‘Z3’) with red, pink and yellow flower petals, respectively, were chosen as plant materials and picked at the full bloom from Hangzhou Administration Garden, China, during the 2014 season. Hangzhou Administration Garden is a national park and concerns with protection of genetic resources of ornamentals including chrysanthemums. It supports our study and offered the authority of sampling these plant materials used in this study.

Three biological replicates (10 g per replicate) of ray florets were frozen in liquid nitrogen immediately after being cut into small pieces and then stored at -80°C for subsequent measurement of anthocyanin content and RNA isolation.

### Anthocyanin contents measurements

Total anthocyanin contents in the ray florets were measured by the pH difference method as described in our previous report [10]. Anthocyanins were extracted from 1 g frozen powdered samples in methanol/0.05% HCl and then absorbance measured in a UV-2550 spectrophotometer (Shimadzu) at 510 and 700 nm. For measurement of anthocyanin in tobacco leaves, frozen leaves were ground to a powder, extracted with methanol/1% HCl overnight at 4°C in the dark and the chlorophyll was then removed with an equal volume of chloroform before measurement of the absorbance at 530 and 657 nm. The relative anthocyanin content in tobacco leaves was calculated on a per g fresh weight basis by ((A530—A657)/mg FW tissue) ×1,000.

### Gene cloning and sequence analysis

A partial CDS (Coding sequence) for one candidate *bHLH* gene related to anthocyanin biosynthesis regulation was isolated from the chrysanthemum ESTs (expressed sequence tags) database (http://www.ncbi.nlm.nih.gov/nucest/term=chrysanthemum). To obtain the full length ORF (Open reading frame), 3′ RACE (Rapid amplification of cDNA ends) and 5′ RACE were performed using the SMART^TM^ RACE cDNA Amplification Kit (Clontech, USA) with primers listed in Table 1. The ORF of this member was predicted by ORF Finder (http://www.ncbi.nlm.nih.gov/gorf/gorf.html) and then verified through PCR with FastStart High Fidelity (Roche, Switzerland) with the primers listed in Table 1.

**Table 1:** Primers used in this research.

Genes	Assays	Primers	Sequences (5'-3')
***CmbHLH1***	QPCR	Forward	CCTCCACCTGGAATCCAGGCTGC
		Reverse	CAGATGGAAGCTCCTCGGCTCAG
	SK	Forward	ATGGTTTCACCGGAGACTACTAC
		Reverse	TTAGGCAACAGGAGGGCGAAGCAC
	AD/BD	Forward	ATCGATACCGAATTCATGGTTTCACCGGAGACTACTAC
		Reverse	ATTGGTACCGGATCCTTAGGCAACAGGAGGGCGAAGCAC
***CmbHLH2***	3'RACE	GSP1	GTGACAGAGAACCAGCAAGCAGCAAC
		GSP2	TGGTGTCGGATTGGTTGGAGAGGCATAC
	5'RACE	GSP1	GTCGCTGCTTCGTGTATGCCTCTCCAAC
		GSP2	TAGTTGCTGCTTGCTGGTTCTCTGTCAC
	QPCR	Forward	GTGAAGGTGAAGGGTATTAGGGGG
		Reverse	CTCTTCAAACGTCCTTCACATACC
	SK	Forward	ATCGATACCGTCGACATGGCTGCCAGCGGACCACCTCG
		Reverse	ATTGGTACCGGGCCCCTAAGGAGATATTATTTGGTTGAT
	AD/BD	Forward	ATGAATTCATGGCTGCCAGCGGACCACCTCG
		Reverse	ATGGATCCCTAAGGAGATATTATTTGGTTGAT
***CmMYB6***	AD/BD	Forward	ATGAATTCATGGGGGAGTACAGAAAAATGAGAC
		Reverse	ATGGATCCGGATTGCAATATCATAGTTGGTCCG
***CmDFR-P***	Y1H	Forward	ATGAGCTCGATGTGATTTTGGTGTTGACTTGG
***AbAi***		Reverse	ATGTCGACGTTGTTTAATCTTGTGGTTTTTGAAG

The *bHLH* member was designated *CmbHLH2* following the first identified member *CmbHLH1* (GenBank KC686698.1) which was chosen as control in this study. The sequence alignment of two *CmbHLHs* and other *bHLH* members related to anthocyanin biosynthesis regulation in other species was performed by CLC Sequence Viewer 6 and Gendoc. Phylogenetic analysis was conducted by MEGA 6.06 [31].

### QPCR (Real-time quantitative PCR) analysis

Total RNA extraction and cDNA synthesis were conducted according to our previous report [10]. RNA extractions were conducted with three biological replicates. The gene expression patterns were analyzed by QPCR analysis following the manufacturers’ instructions using Ssofast EvaGreen supermix (Bio-rad, USA). The QPCR primers for the two *bHLH* members are listed in Table 1, while those for anthocyanin biosynthetic genes were as referred to by Huang et al. [11]. All of the primers specificities were verified by both melting curves and QPCR products sequencing [32]. *CmACT* (GenBank AB770471) was used as a reference gene to evaluate the expression of genes of interest. No-template reactions were set as negative controls for each gene.

### Dual luciferase assay

Dual luciferase assay, using pGreenII0029 62-SK and pGreenII 0800-LUC vectors, has been widely used to study the transcription regulating effects of transcription factors on their target promoters [10, 33–35]. The ORFs of *CmbHLH1* and *CmbHLH2* were amplified with the primers listed in Table 1 and recombined into pGreenII0029 62-SK vectors’ MCS (multiple cloning sites), respectively. The recombinant plasmids of *CmMYB6-*SK and *CmDFR* promoter-LUC in this study were constructed by Liu et al. [10]. Each of these recombinant plasmids was transferred into *Agrobacterium tumefaciens* GV3101 (MP90) individually.

GV3101 (MP90) containing *CmbHLH*-SK or/and *CmMYB6*-SK were mixed with *CmDFR* promoter-LUC at 10:1 ratio before infiltration into tobacco leaves (*Nicotiana benthamiana*). The ratio of enzyme activities of firefly luciferase (*CmDFR*::LUC) to renilla luciferase (35S::REN) was analyzed by Dual-Luciferase Reporter Assay System (Promega, USA) with Modulus Luminometer (Promega, USA) following the manufacturers’ instructions. Three independent experiments were carried out with at least four biological replicates for each.

### Transient over-expression

Tobacco leaves (*N*. *tabacum*) were chosen for transient over-expression analysis of *CmbHLH2*. GV3101 (MP90) containing *CmbHLH1*-SK or *CmbHLH2*-SK was mixed with *CmMYB6*-SK or empty vector at 1:1 ratio before infiltration into tobacco leaves. The infiltrated patches of tobacco leaves were photographed and anthocyanin contents measured eight days after infiltration. Three independent experiments were carried out with three biological replicates for each combination.

### Yeast hybridization

Yeast one-/two-hybrid (Y1H/Y2H) system were used to study protein-DNA or protein-protein interaction, respectively, according to the manufacturers’ instruction and as described in our previous publications [34, 35].

The *CmDFR* promoter was integrated into the genome of the Y1H Gold yeast strain by homologous recombination to generate the *CmDFR*-pAbAi bait strain after it was cloned into the pAbAi vector. The potential DNA-binding proteins, CmMYB6, CmbHLH1 or CmbHLH2, were expressed as fusion proteins containing the yeast GAL4 transcription activation domain (AD-CmMYB6, AD-CmbHLH1 or AD-CmbHLH2) recombined into pGADT7 vector, respectively, with the primers listed in Table 1. Then the minimal inhibitory concentration of Aureobasidin A for the bait strain was tested on SD/-Ura and SD/-Ura+AbA^x^ media before testing the protein-DNA interactions which were screened on SD/-Leu media containing the minimal concentration of AbA antibiotic.

The ORFs of *CmMYB6*, *CmbHLH1* and *CmbHLH2* were recombined into pGBKT7 vector to express the bait proteins BD-CmMYB6, BD-CmbHLH1 or BD-CmbHLH2. Then all of these bait proteins were transformed into Y2H yeast stain, individually, to test their auto-activation on SD/-Trp media with X-α-Gal and AbA antibiotic background. Subsequently, the protein-protein interaction were detected by co-transforming the prey and bait plasmids into Y2H yeast strain and screening for growth of colonies on quadruple SD/-Ade/-His/-Leu/-Trp and SD/-Ade/-His/-Leu/-Trp+ X-α-Gal+ AbA media.

## Results

### *CmbHLH2* predicted to regulate anthocyanin biosynthesis

**Fig 1:**
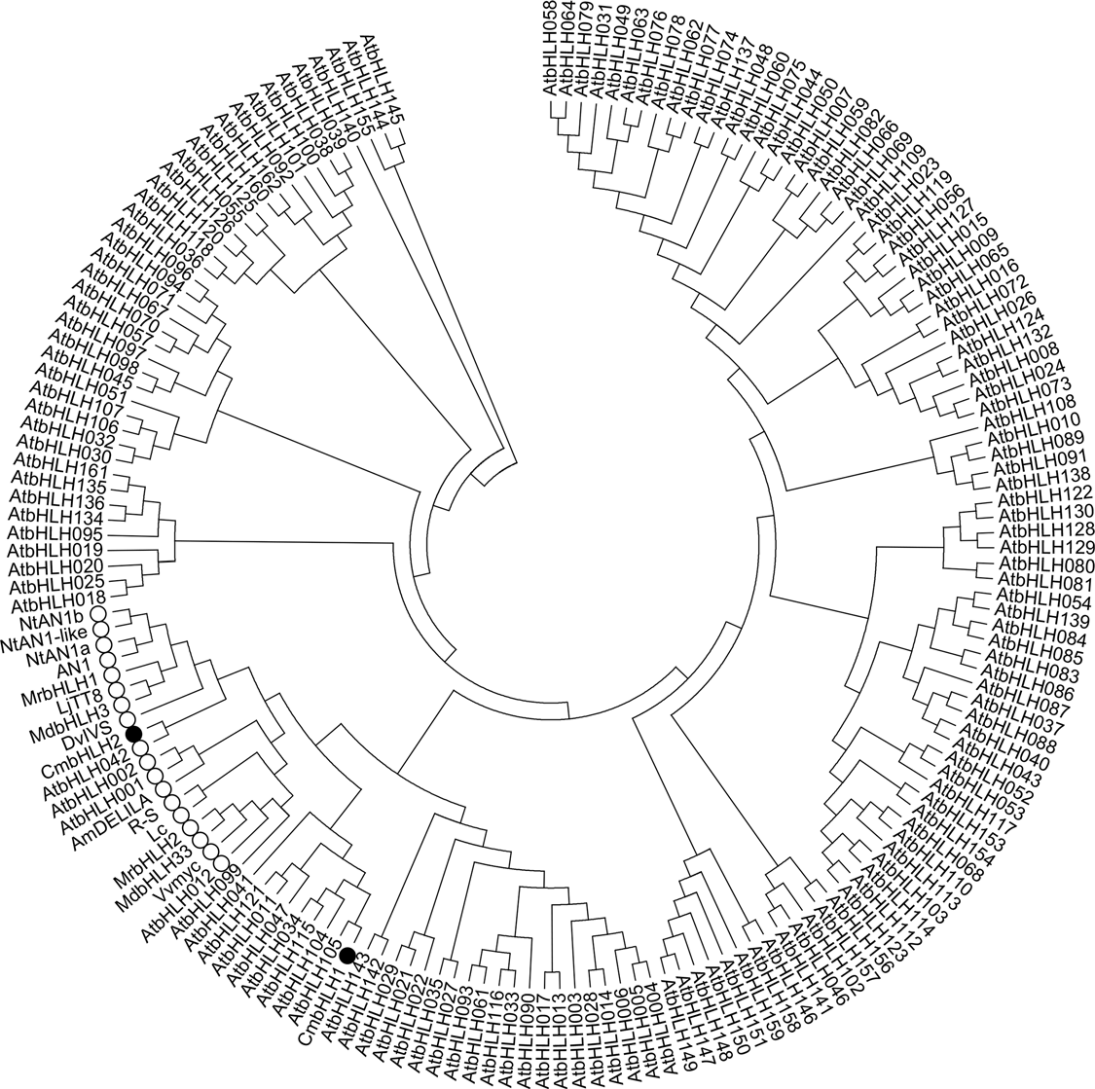
Phylogenetic analysis of 178 *bHLH* members including 162 from *Arabidopsis*, 14 from other plants, and 2 from chrysanthemum. The two *bHLHs* from chrysanthemum are marked with solid circles, while those related to anthocyanin biosynthesis regulation in *Arabidopsis* and other species are marked with open circles. Genes IDs are as follows: NtAn1b (*Nicotiana tabacum*, GenBank accession number AEE99258), NtAN1-like (*N*. *tomentosiformis*, AEE99260), NtAn1a (*N*. *tabacum*, AEE99257), AN1 (*Petunia hybrid*, AF260918), MrbHLH1 (*Myrica rubra*, JX629461), LjTT8 (*Lotus japonicus*, BAH28881), MdbHLH3 (*Malus domestica*, HM122458), DvIVS (*Dahlia variabilis*, AB601005), AmDELILA (*Antirrhinum majus*, AAA32663), R-S (*Zea mays*, X15806), L-c (*Z*. *mays*, NM001111869), MrbHLH2 (*M*. *rubra*, JX629462), MdbHLH33 (*M*. *domestica*, DQ266451), VvMYC (*Vitis vinifera*, EU447172).

Based on the similarities with *bHLHs* verified as being related to anthocyanin biosynthesis in other species, one partial sequence *bHLH* member was mined from the chrysanthemum EST database. The full ORF, which was obtained by RACE and named as *CmbHLH2* (GenBank KT724056), contained 1842 nucleotides and encoded 614 amino acids. A second *bHLH* member isolated from chrysanthemum, *CmbHLH1*, which was reported to be involved in the response to iron deficiency [30] was also selected. Phylogenetic analysis of these two members and 174 *bHLHs* including 158 from *Arabidopsis* and 14 from other plants, showed that *CmbHLH2* was clustered in the subgroup containing sequences related to the regulation of anthocyanin biosynthesis, while *CmbHLH1* was in a different subgroup (Fig 1).

The conserved basic helix-loop-helix (bHLH) domain containing approximately 60 basic amino acids was present in both *CmbHLH1* and *CmbHLH2* (S1 Fig). However, there were some differences between these two bHLH domains. The N-terminus of *CmbHLH2* bHLH included a highly conserved HER motif (His5-Glu9-Arg13) which was also found in other *bHLH* members related to anthocyanin biosynthesis in other species and participated in binding the E-box (CANNTG) DNA motif (S1 Fig). Furthermore, the MYB interaction region (MIR) including box11, box13 and box18, which is able to bind the MYB to form a transcription complex, was located in the N-terminal region of the CmbHLH2 amino acid sequence (S1 Fig). However, these two characteristic features were not found in the CmbHLH1 sequence (S1 Fig).

### Differences in ability to accumulate Anthocyanin among three chrysanthemum cultivars

**Fig 2:**
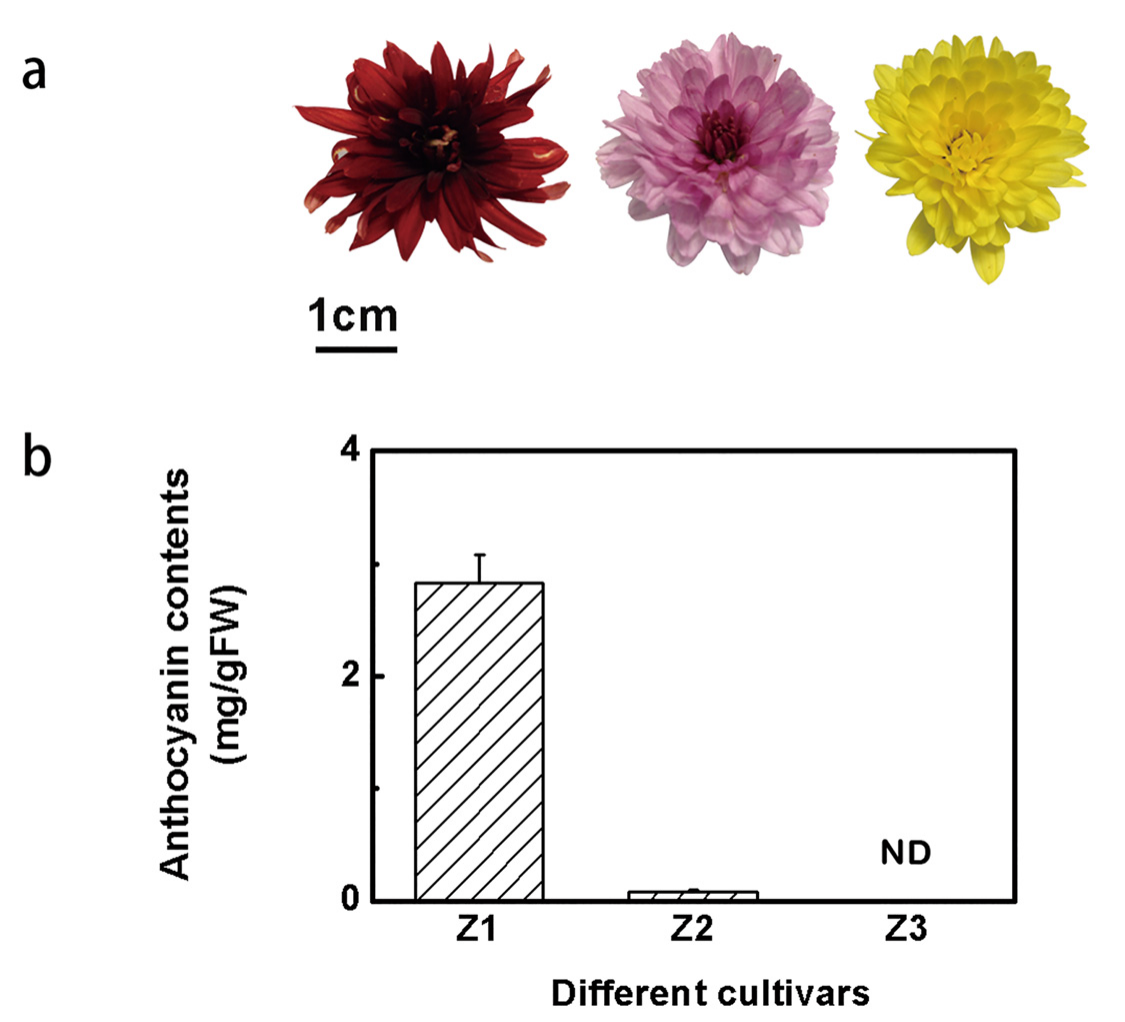
The anthocyanin contents of flowers of three different chrysanthemum cultivars. (a) The photographs of three chrysanthemum cultivars, named ‘Z1’, ‘Z2’ and ‘Z3’, respectively, where the bar represents 1 cm. (b) Anthocyanin contents in flower petals from Z1, Z2 and Z3. The vertical bars represent S.E. of three biological replicates.

Three chrysanthemum cultivars used in this study had different flower colors (Fig 2A). Further measurement indicated that anthocyanin contents varied among them. ‘Z1’, with deep red ray florets, accumulated 2.83 mg/gFW anthocyanin compared with 0.08 mg/gFW in ‘Z2’ which had pink flower colors (Fig 2B). No anthocyanin was detected in ‘Z3’, which had yellow flowers (Fig 2B). This suggested that difference in anthocyanin contents was the main reason for the different colors of ray florets of these three chrysanthemum flower cultivars.

### *CmbHLH2* expression positively correlated with anthocyanin accumulation

**Fig 3:**
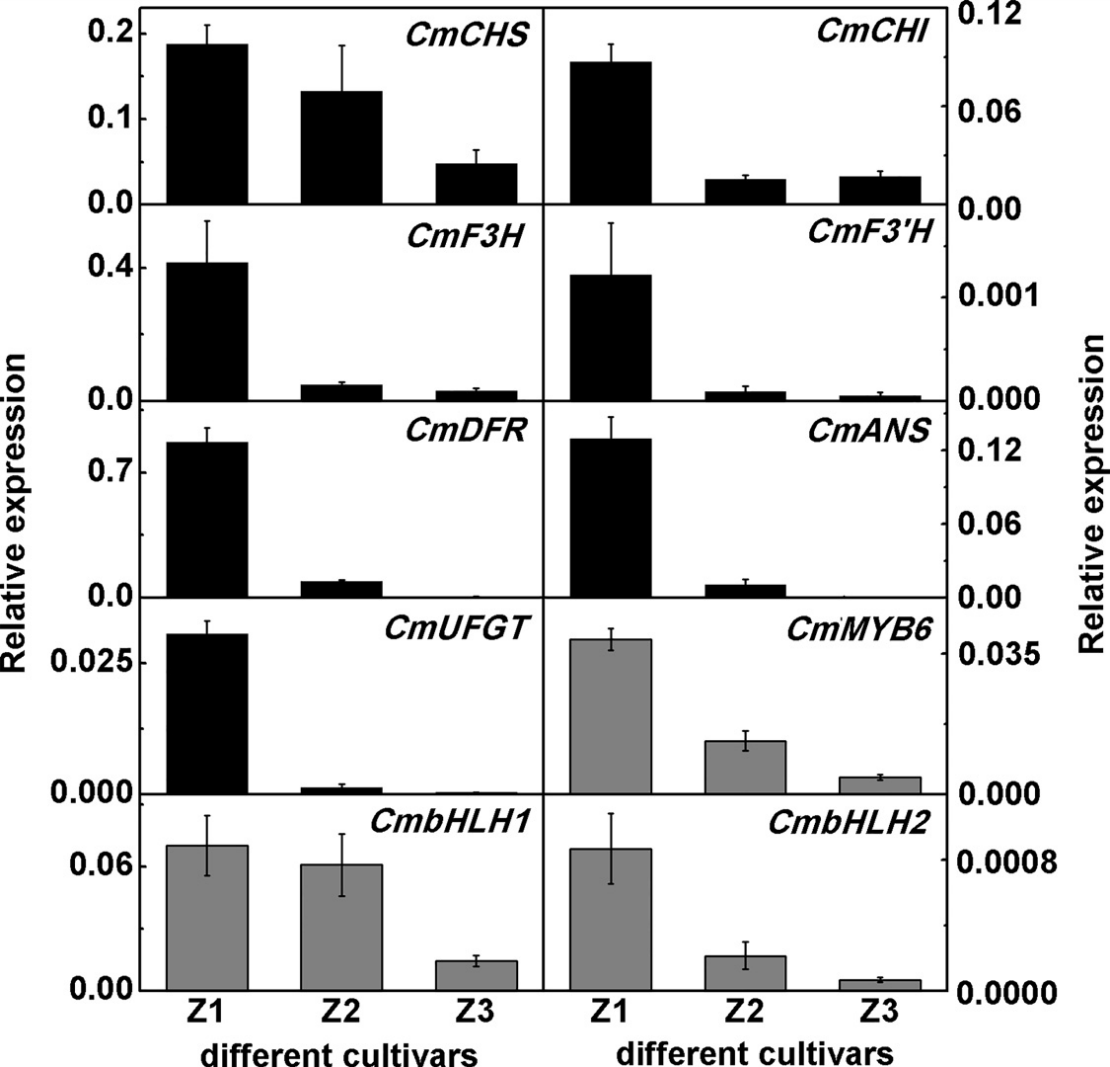
The transcript levels of seven anthocyanin biosynthetic genes and three TFs in the ray florets of three chrysanthemum cultivars used in this study. The *CmACT* gene was used to normalize expression of the genes. Non-template reactions were set as the negative control for each gene. The vertical bars represent S.E. of three biological replicates.

Accumulation of mRNAs for three TFs, *CmMYB6*, *CmbHLH1* and *CmbHLH2*, and seven anthocyanin biosynthetic genes in the ray florets of three cultivars were analyzed using QPCR. All biosynthetic genes were expressed at the highest level in ‘Z1’ and were positively correlated with anthocyanin accumulation in these three cultivars, although the transcriptional level of *CmCHI* (Chalcone isomerase) was similar in ‘Z2’ and ‘Z3’ (Fig 3). The transcript levels of *CmDFR* and *CmUFGT* (UDP flavonoid glucosyl transferase) were very low in ‘Z3’ compared with those in ‘Z1’, and *CmANS* (Anthocyanidin synthase) was undetectable in ‘Z3’ (Fig 3). Accumulation of mRNA for each of the three TFs was highest in ‘Z1’ and lowest in ‘Z3’ (Fig 3) as found for the biosynthetic genes.

### *CmbHLH2* stimulated *CmDFR* promoter activity when coupled with *CmMYB6*

**Fig 4:**
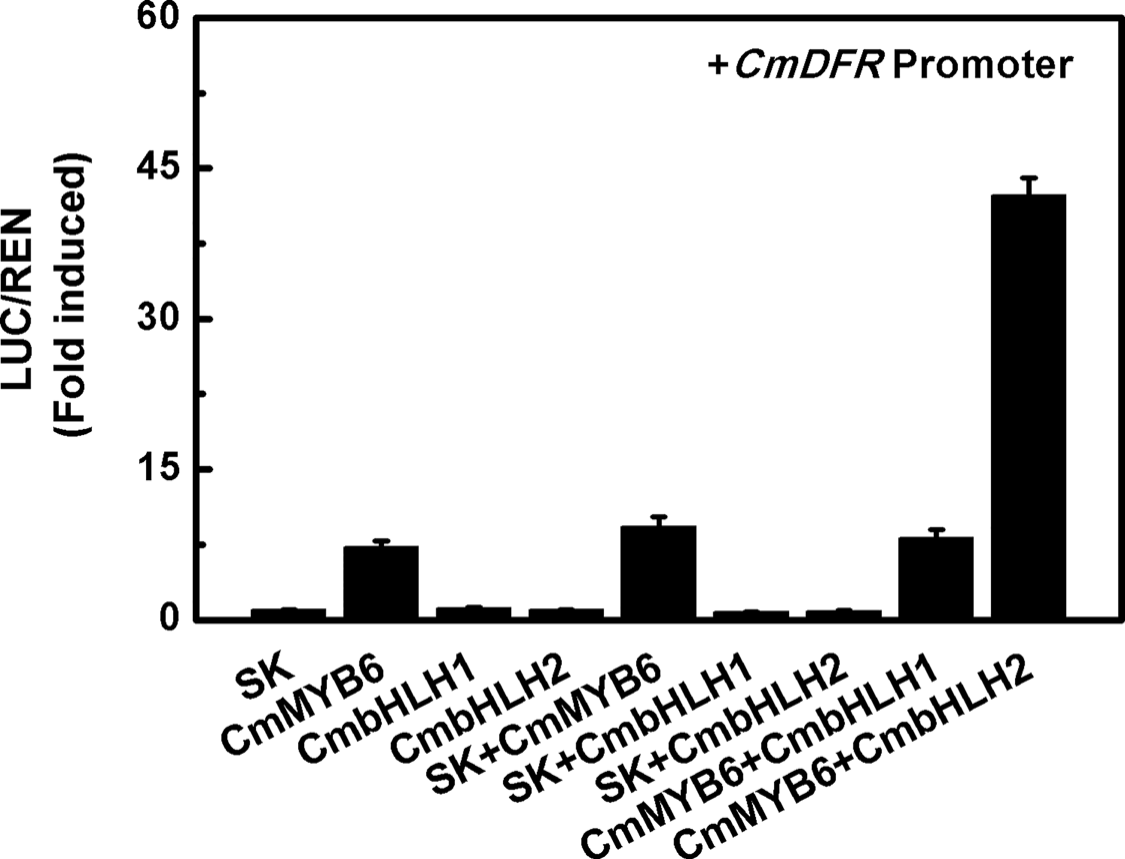
*In vivo* interactions between *CmMYB6*, *CmbHLHs* and the *CmDFR* promoter were revealed by dual luciferase assays in tobacco leaves. Error bars are the S.E. of three independent experiments with at least four replicates.

To predict the transcriptional regulating roles of *CmbHLH1* and *CmbHLH2*, dual luciferase assay with the *CmDFR* promoter were used in this study. Based on the results, *CmbHLH1* or *CmbHLH2* singly had no effect on the activity of *CmDFR* promoter compared to the empty vector, activity of which was set as one, whereas *CmMYB6* induced an approximately 8-fold increase in *CmDFR* promoter activity (Fig 4). However, when co-expressed with *CmMYB6*, *CmbHLH2* showed a significant synergistic effect and *CmDFR* promoter activity was stimulated over 40-fold (Fig 4). *CmbHLH1*, on the other hand, was unable to up-regulate the activity of *CmDFR* promoter when co-expressed either with empty vector or with *CmMYB6* (Fig 4). This indicated that *CmbHLH2*, but not *CmbHLH1*, has the ability to regulate anthocyanin biosynthesis.

### *CmbHLH2* directly bound to *CmDFR* promoter

**Fig 5:**
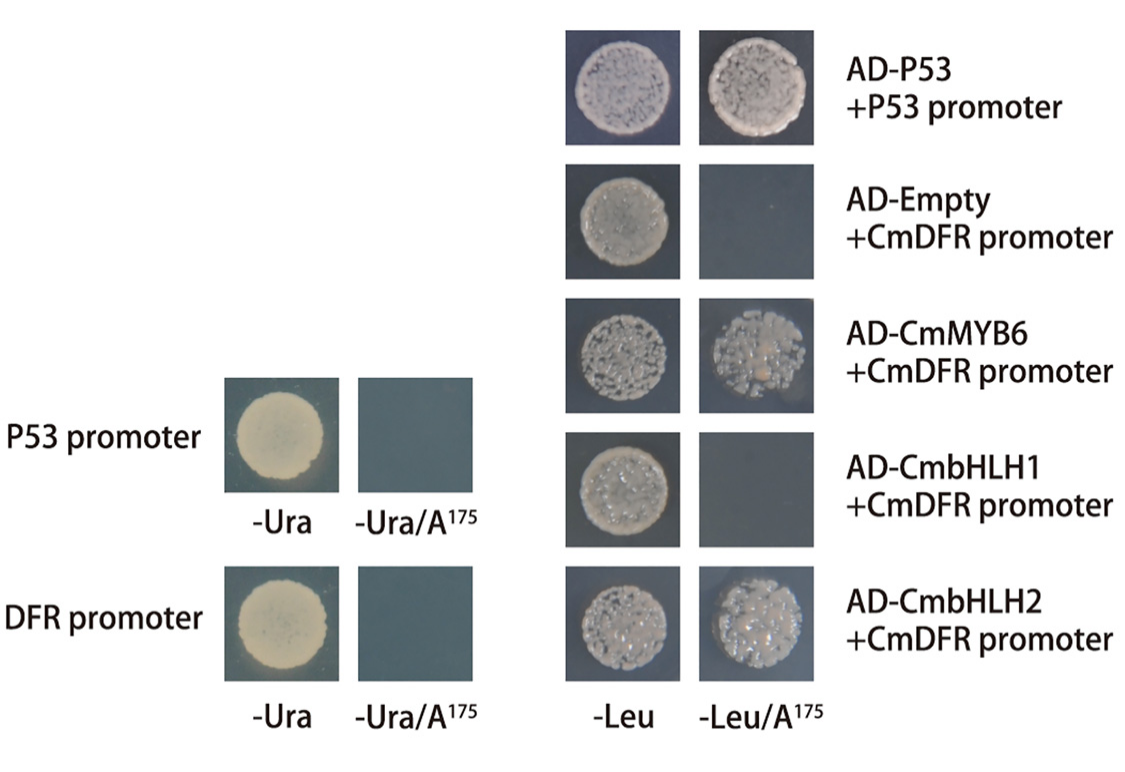
Characterization of the interaction of three TFs with the *CmDFR* promoter by yeast one-hybrid assays. P53 and its promoter supplied with the kit were used as a positive control to verify the stability of these assays. The auto-activity of *CmDFR* promoter bait stain was tested on SD media lacking Ura in presence of AbA. *CmbHLH1*, *CmbHLH2* and *CmMYB6* were fused into pGADT7 (AD) and transformed separately into the bait stain. The binding was screened on SD media lacking Leu in presence of AbA^175^.

The relationship between CmbHLH2 and the *CmDFR* promoter was further analyzed by yeast one-hybrid assay. Successful generation of the *CmDFR*-pAbAi bait strain was confirmed by screening colony growth on SD/-Ura media and auto-activation was inhibited with 175 ng/ml AbA antibiotic (Fig 5). When the bait strains were transformed with the fusion protein AD-CmMYB6, AD-CmbHLH1 or AD-CmbHLH2, which has the activation domain, different colony growing abilities were detected on the testing media. Strains transformed with AD-CmMYB6 or AD-CmbHLH2 could grow on SD/-Ura+AbA^175^, while those with either empty vector or AD-CmbHLH1 could not (Fig 5).

### *CmbHLH2* interacted with *CmMYB6* to form a transcriptional complex

**Fig 6:**
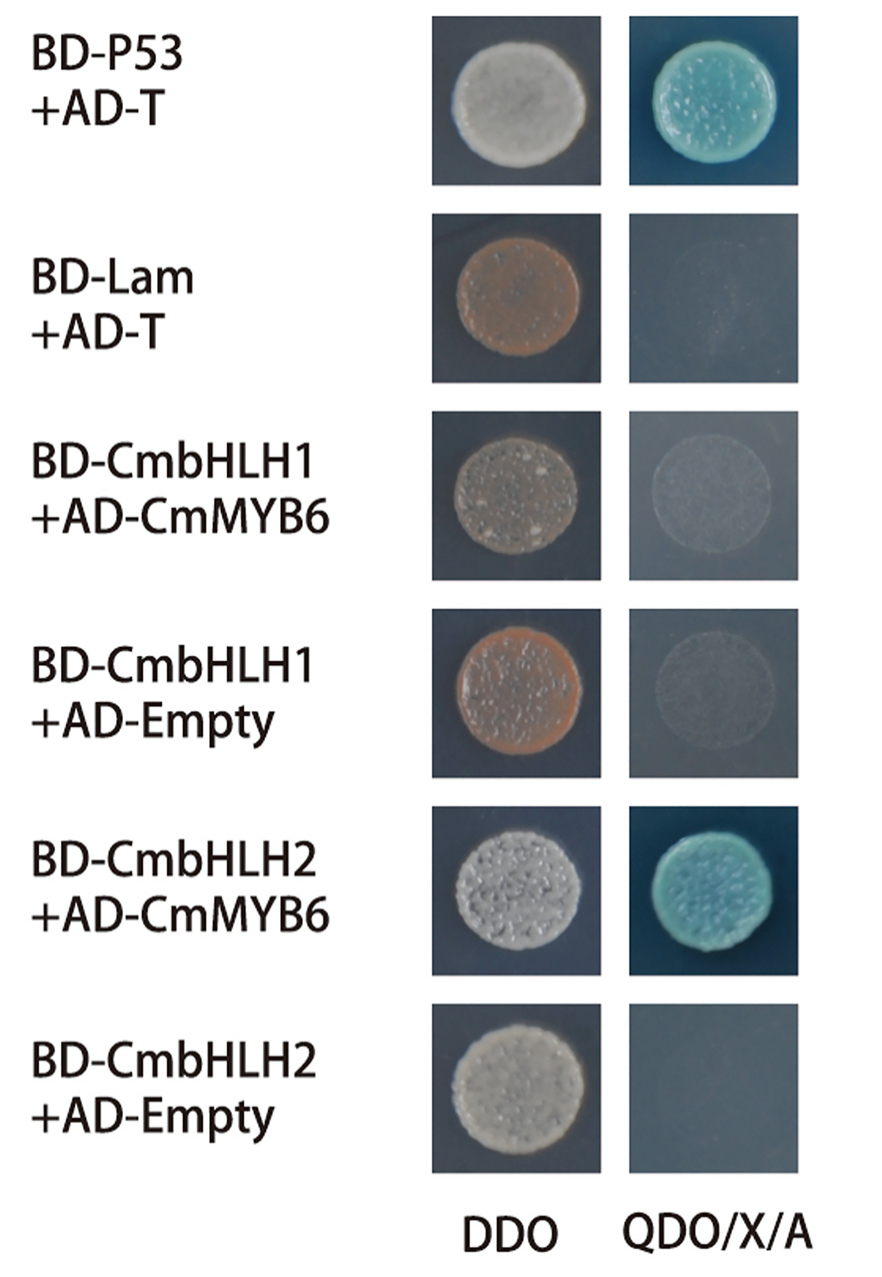
Protein-protein interactions between *CmbHLHs* and *CmMYB6* studied by yeast two-hybrid assay. The yeast strain was co-transformed with the indicated combinations of *CmbHLH1* or *CmbHLH2* fused into pGBKT7 (BD) and *CmMYB6* fused into pGADT7 (AD). BD-p53 and AD-T were used as positive controls, while BD-Lam and AD-T were used as negative controls. Protein-protein interactions were detected on DDO (SD media lacking Leu and Trp) and QDO/X/A (SD media lacking Leu, Trp, His and Ade, with AbA and X-α-gal) media.

To study the mode of interaction between CmbHLH2 and CmMYB6 proteins during the transcriptional regulation of *CmDFR* promoter, Y2H assay were carried out. Based on the testing results, the auto-activation of BD-CmbHLH1 and BD-CmbHLH2 were inhibited with 100 ng/ml and 400 ng/ml AbA, respectively (S2 Fig). However, auto-activation of BD-CmMYB6 could not be inhibited even with 1000 ng/ml AbA (S2 Fig), thus CmMYB6 was used as the prey protein and CmbHLH1 or CmbHLH2 as bait proteins in subsequent experiments. Protein-protein interaction between CmMYB6 and CmbHLH2 was indicated by growth of colonies containing both AD-CmMYB6 and BD-CmbHLH2 proteins on SD/-Ade/-His/-Leu/-Trp/+X-α-Gal/+AbA screening media (Fig 6).

### *CmbHLH2* together with *CmMYB6* triggered anthocyanin accumulation in tobacco leaves

**Fig 7:**
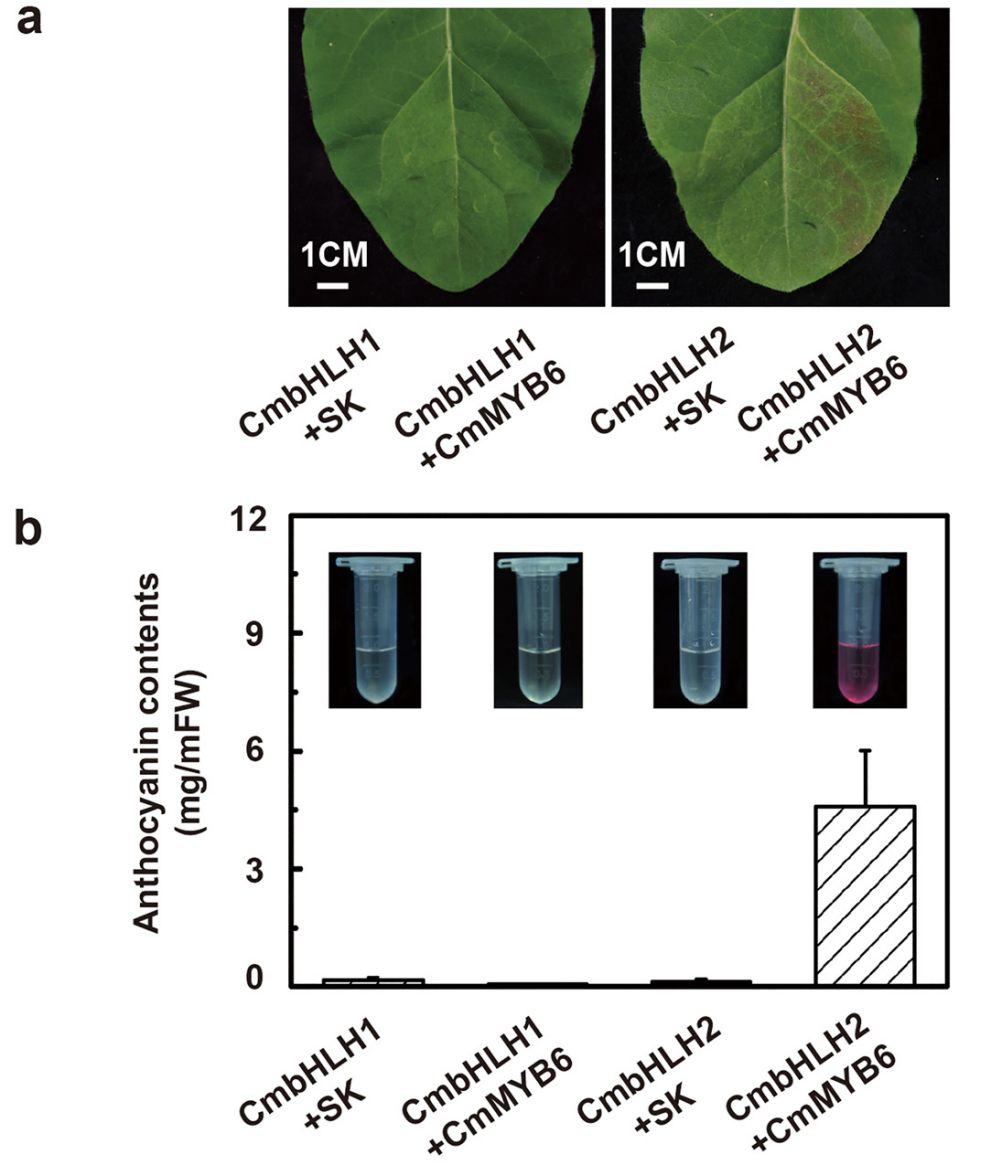
Transient over-expression of *CmMYB6* and *CmbHLH1* or *CmbHLH2* carried out in tobacco leaves. (a) Photographs taken 8 days after the infiltration. (b) Analysis of anthocyanin contents of tobacco leaves 8 days after infiltration with the TFs. Each experiment was carried out with three biological repeats and the error bars represent the S.E. of these replicate reactions.

To confirm the role of *CmbHLH2* in the regulation of anthocyanin biosynthesis, a transient expression assay was performed in tobacco leaves. Although *CmMYB6* could stimulate the activity of the *CmDFR* promoter (Fig 4), anthocyanin accumulation could not be triggered by a single *CmMYB6* member (S3 Fig). Similarly, *CmbHLH1* or *CmbHLH2* singly could not regulate anthocyanin biosynthesis in tobacco leaves (Fig 7). Only the patches infiltrated with both *CmMYB6* and *CmbHLH2* had the ability to produce a dramatic accumulation of anthocyanin, of up to 5.0 mg/gFW on the 8^th^ day after infiltration (Fig 7). Based on these results, *CmbHLH2* is the crucial and essential member of the MBW transcription complex regulating anthocyanin biosynthesis together with *CmMYB6* in chrysanthemum.

## Discussion

Chrysanthemum is an important and popular ornamental crop worldwide. During the long breeding history, lots of cultivars have been generated and cultivated [36]. The agricultural traits vary significantly among these cultivars. The three different cultivars ‘Z1’, ‘Z2’ and ‘Z3’ were chosen as plant materials because of their high similarities except for the flower colors. Most of the agricultural traits of these three cultivars, such as plant height and type and size of, leaves and, flowers are similar. Furthermore, the full blooming dates of ‘Z1’, ‘Z2’ and ‘Z3’ are almost the same.

*CmbHLH2* was the first confirmed bHLH member related to anthocyanin biosynthesis regulation in chrysanthemum. Its sequence alignment showed conserved among different cultivars of chrysanthemum, while suggested variable compared to members related to anthocyanin biosynthesis regulation in other species. The full length sequence of *CmbHLH2* was obtained from ‘Z1’ cDNA by means of RACE using the reference sequence mined from the EST database of the ‘Zhongshanzigui’ cultivar [37]. Subsequently, comparison of *CmbHLH2* ORFs in different cultivars, including ‘Z2’, ‘Z3’ and ‘Amadea’ which is a cut chrysanthemum cultivar with purple flower colors [10], were carried out through gene cloning and sequence alignments. However, no significant differences were found in these sequences (data not shown). This indicated the strong conservation of *CmbHLH2* ORF in different chrysanthemum cultivars studied. Sequence comparisons with related genes from other species, however, gave low identities. For example, *CmbHLH2* shared 64% identities with *DvIVS* (*Dahlia pinnata*, [28]), 48% with *PhAN1* (*Petunia*×*hybrida*, [26]) and 59% with *AtTT8* (*AtbHLH042*, *Arabidopsis thaliana*, [27]). Although all of these *bHLH* members play essential roles in the regulation of anthocyanin biosynthesis, the similarities in their sequences were restricted to the MIR (box 11, box 13 and box 18) and bHLH domain which are responsible for the interaction with *MYB* partners and binding to the target gene promoters, respectively [20]. Thus, these sequences could be used to predict the potential role of *bHLH* members in anthocyanin biosynthesis regulation.

TFs regulate the target genes through binding to the *cis-*elements located in the gene promoters. The *bHLH* encoded proteins are known to bind to the E/G-box (CANNTG/CACGTG), but the MRE (*MYB* recognizing elements) motifs vary greatly in different gene promoters [7]. For example, there were as many as nine different kinds of MRE motifs found in *MrDFR1* and *MrUFGT* promoters involved in anthocyanin biosynthesis in Chinese bayberry [19]. *Cis*-elements recognized by MYB or bHLH have been found in the *CmDFR* promoter sequence, for example, MYBPZM (CCWACC), MYB1AT (WAACCA) and MYBATRD22 (CACATG), recognized by MYB, and E-box (CANNTG) and ACEs (ACGT containing elements), recognized by bHLH [10]. In this study, both *CmMYB6* and *CmbHLH2* could bind to the *CmDFR* promoter as tested by Y1H assay (Fig 5). However, whether they recognizing the exact *cis*-elements predicted in *CmDFR* promoter remains unclear and need further verification. Furthermore, the last 26 amino acids located before the *CmbHLH2* termination codon played an important role in binding the *CmDFR* promoter during anthocyanin biosynthesis regulation. In our study, another bHLH member which showed 100% identities to *CmbHLH2* sequence except for the last 26 amino acids, had no ability to bind the *CmDFR* promoter based on Y1H assay. No anthocyanin accumulation could be detected in tobacco leaves transient over-expressed both this bHLH member and *CmMYB6*, although it could interact with *CmMYB6* in Y2H system (data not showed). However, the novel characteristic of this bHLH member in anthocyanin biosynthesis in chrysanthemum still needs further study.

Interactions between TFs are the central to life processes in bionts and the interaction among MYB, bHLH and WDR have been well characterized in all angiosperms analyzed so far [7]. Two MBW complexes related to flavonoid biosynthesis regulation, such as PhAN2-PhAN1-PhAN11 in petunia [26] and AtTT2-AtTT8-AtTTG1 in *Arabidopsis* [38] have been characterized. The interaction between CmMYB6 and CmbHLH2 indicated by Y2H test in this study (Fig 6) was in some respects similar to the AtTT2/AtTT8 physical interaction to form a TF complex active during the accumulation of PAs (proanthocyanins) in *Arabidopsis* [38]. Based on this evidence, it is suggested that CmMYB6 and CmbHLH2 form a binary complex through physically interacting with each other to regulate expression of *CmDFR* during anthocyanin biosynthesis in chrysanthemum.

Based on previous results, MYB proteins frequently determine the involvement of MBW complexes in specific pathways, although the specificity of the MBW complexes for different targets most probably relies on both MYB and bHLH, as well as on other regulators [7]. Our results show that anthocyanin biosynthesis was triggered only when CmMYB6 and CmbHLH2 were co-expressed in tobacco leaves (Fig 7). It is unclear, however, whether the endogenous WDR member(s) in tobacco leaves participated this process the possible role(s) of endogenous WDR member(s) in anthocyanin accumulation in chrysanthemum requires further study.

## Conclusions

Differences in flower colors between ‘Z1’, ‘Z2’ and ‘Z3’ were due to their differing abilities to accumulate anthocyanins. Expression of all seven biosynthetic genes, as well as TFs *CmbHLH2* and *CmMYB6*, was positively correlated with the anthocyanin content in different cultivars. The IIIf subgroup member, *CmbHLH2*, contained a conserved bHLH domain and MIR regions and stimulated *CmDFR* promoter activity up to 40-fold through physical interaction with *CmMYB6*, indicating it is a partner in the transcription complex controlling expression of anthocyanin biosynthesis genes in developing chrysanthemum flowers.

## Supporting Information

S1 Fig Structure of *bHLHs* involved in regulation of anthocyanin biosynthesis, with sequences of MIR and BHLH regions. Three conserved boxes, including box11, 18 and 13, found in these *bHLHs* play essential roles in the interaction with *MYBs* transcription factors leading to enhanced transcription of anthocyanin biosynthesis genes. Amino acid residues (H-E-R) at position 5, 9 and 13 of the bHLH domains are critical for DNA binding.
(TIF)

S2 Fig Testing the auto-activation of three bait proteins used yeast two-hybrid assays. Three TFs, *CmMYB6*, *CmbHLH1* and *CmbHLH2*, were cloned separately into pGBKT7 (BD). Auto-activation was screened on SD/-Trp media with X-α-Gal and AbA antibiotic background.
(TIF)

S3 Fig Transient over-expressions of *CmMYB6* and *CmbHLH1* or *CmbHLH2* were carried out in tobacco leaves. The full ORF of each gene was cloned into pGreenII0029 62-SK and electroporated into *Agrobacterium tumefaciens* GV3101 (MP90). Different combinations of these *Agrobacterium tumefaciens* were infiltrated into tobacco leaves. Photographs were taken 8 days later. Each experiment was carried out with three biological repeats.
(TIF)

## Abbreviations

AbA: Aureobasidin
ANS: Anthocyanidin synthase
bHLH: basic helix-loop-helix
CDS: Coding sequence
CHI: Chalcone isomerase
CHS: Chalcone synthase
DFR: Dihydroflavonol 4-reductase
ESTs: Expressed sequence tags
F3H: Flavanone 3-hydroxylase
F3'H: Flavanone 3'-hydroxylase
LUC: Firefly luciferase
MBW: MYB-bHLH-WD40
MCS: Multiple cloning sites
MRE: MYB recognizing elements
MIR: MYB interaction region
ORF: Open reading frame
QPCR: Real-time quantitative PCR
RACE: Rapid amplification of cDNA ends
REN: Renilla luciferase
SD: Synthetic dropout
TFs: Transcription factors
UFGT: UDP flavonoid glucosyl transferase
UTR: Untranslated region
Y1H: Yeast one-hybrid
Y2H: Yeast two-hybrid
