# Supplementary figures and images for: A Novel bHLH Transcription Factor Involved in Regulating Anthocyanin Biosynthesis in Chrysanthemums (Chrysanthemum morifolium Ramat.)
Source: PLoS One. 2015 Nov 30;10(11):e0143892. doi: 10.1371/journal.pone.0143892 (PMC4664390; doi:10.1371/journal.pone.0143892)

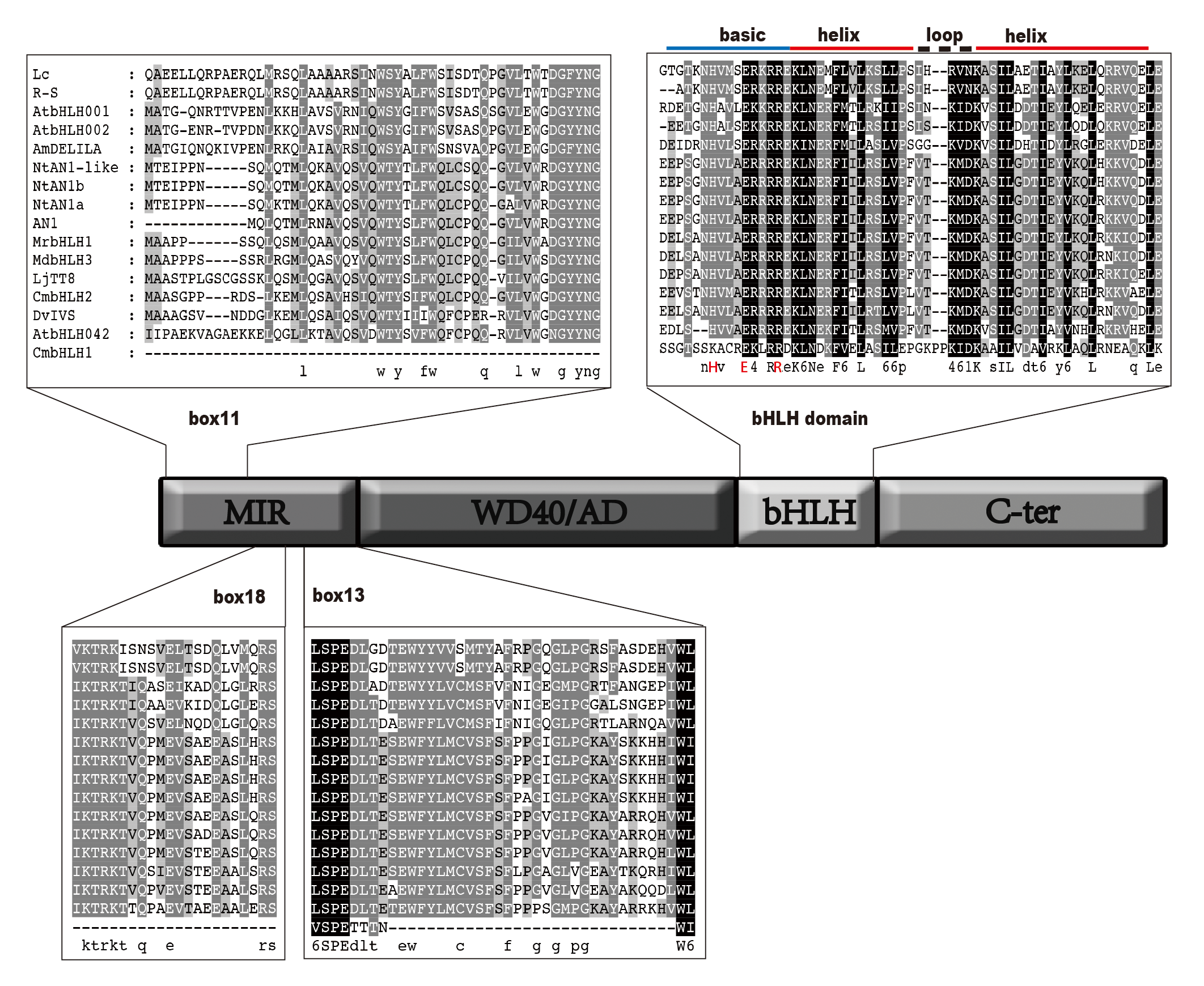

Supplement: S1 Fig — Three conserved boxes, including box11, 18 and 13, found in these bHLHs play essential roles in the interaction with MYBs transcription factors leading to enhanced transcription of anthocyanin biosynthesis genes. Amino acid residues (H-E-R) at position 5, 9 and 13 of the bHLH domains are critical for DNA binding. (TIF) [file pone.0143892.s001.tif]

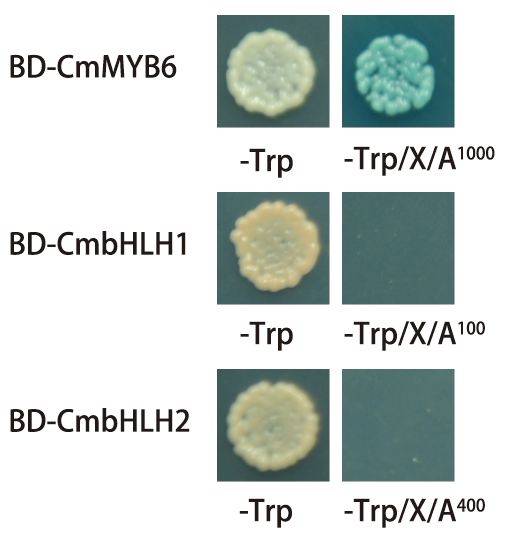

Supplement: S2 Fig — Three TFs, CmMYB6, CmbHLH1 and CmbHLH2, were cloned separately into pGBKT7 (BD). Auto-activation was screened on SD/-Trp media with X-α-Gal and AbA antibiotic background. (TIF) [file pone.0143892.s002.tif]

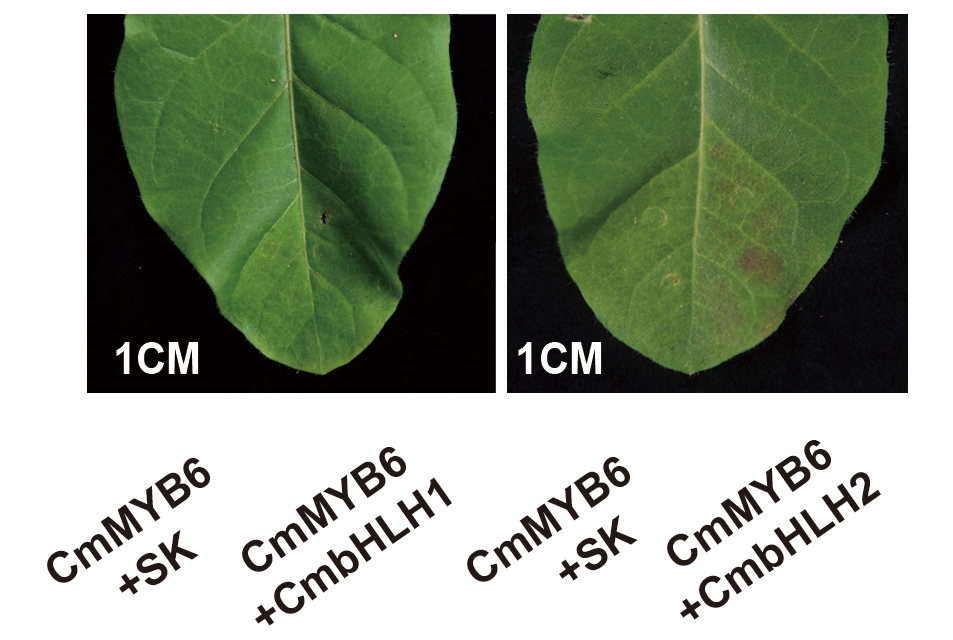

Supplement: S3 Fig — The full ORF of each gene was cloned into pGreenII0029 62-SK and electroporated into Agrobacterium tumefaciens GV3101 (MP90). Different combinations of these Agrobacterium tumefaciens were infiltrated into tobacco leaves. Photographs were taken 8 days later. Each experiment was carried out with three biological repeats. (TIF) [file pone.0143892.s003.tif]
